# Host Deception: Predaceous Fungus, *Esteya vermicola*, Entices Pine Wood Nematode by Mimicking the Scent of Pine Tree for Nutrient

**DOI:** 10.1371/journal.pone.0071676

**Published:** 2013-08-19

**Authors:** Feng Lin, Jianling Ye, Huaguang Wang, Aijun Zhang, Boguang Zhao

**Affiliations:** 1 Department of Forest Protection, Nanjing Forestry University, Nanjing, People’s Republic of China; 2 Animal, Plant and Food Inspection Center, Jiangsu Entry-Exit Inspection and Quarantine Bureau, Nanjing, People’s Republic of China; 3 Invasive Insect Biocontrol and Behavior Laboratory, Agricultural Research Service, United States Department of Agriculture, Beltsville, Maryland, United States of America; Harvard University, United States of America

## Abstract

**Background:**

A nematophagous fungus, *Esteya vermicola*, is recorded as the first endoparasitic fungus of pine wood nematode (PWN), *Bursaphelenchus xylophilus*, in last century. *E. vermicola* exhibited high infectivity toward PWN in the laboratory conditions and conidia spraying of this fungus on Japanese red pine, *Pinus densiflora*, seedlings in the field protected the pine trees from pine wilt disease to some extent, indicating that it is a potential bio-control agent against PWN. Previous research had demonstrated that the living fungal mycelia of *E. vermicola* continuously produced certain volatile organic compounds (VOCs), which were responsible for the PWN attraction. However, identity of these VOCs remains unknown.

**Methodology/Principal Findings:**

In this study, we report the identification of α-pinene, β-pinene, and camphor produced by living mycelia of *E. vermicola*, the same volatile compounds emitted from PWN host pine tree, as the major VOCs for PWN attraction using gas chromatography-mass spectrometry (GC-MS). In addition, we also confirmed the host deception behavior of *E. vermicola* to PWN by using synthetic VOCs in a straightforward laboratory bioassay.

**Conclusions/Significance:**

This research result has demonstrated that the endoparasitic nematophagous fungus, *E. vermicola*, mimics the scent of PWN host pine tree to entice PWN for the nutrient. The identification of the attractive VOCs emitted from the fungus *E. vermicola* is of significance in better understanding parasitic mechanism of the fungus and the co-evolution in the two organisms and will aid management of the pine wilt disease.

## Introduction

*Esteya vermicola* was recorded as the first endoparasitic fungus of the pine wood nematode (PWN), *Bursaphelenchus xylophilus* [1], and exhibited high infectivity *in* *vitro* [2], [3]. The lunate conidia of *E. vermicola* are adhesive and can adhere to the cuticle of PWN, causing subsequent infection. This predaceous fungus consumes the contents of the infected nematode’s body, grows out from its cadaver, and then produces new lunate conidia for the next infection cycle [3]. During a survey of nematophagous fungi in Korea, a novel endoparasitic fungal strain, CNU 120806, was isolated from infected nematodes in forest soil and identified as a rare hyphomycete. Results of taxonomy and molecular phylogenetic analyses showed that CNU120806 was a new strain of *E. vermicola* [3]. *E. vermicola* CNU 120806 also exhibited high infectivity toward PWN in the laboratory conditions. Conidia spraying of *E. vermicola* onto four-year-old *Pinus densiflora* seedlings in the field showed promising potential to be used as a bio-control agent against PWN [2], [3]. Wang et al. demonstrated that the living mycelia and exudative substances of *E. vermicola* were attractive to PWN [4]. Further experiments revealed that the attractive substances from *E. vermicola* consisted of volatile and non-volatile compounds [4]. We hypothesized that these attractive volatile compounds should be related to the PWN hosts. Present research was focused on the identification of volatile organic compounds (VOCs) that were responsible for PWN attraction from *E. vermicola* CNU 120806 using gas chromatography-mass spectrometry (GC-MS).

## Materials and Methods

### Nematodes

PWNs were cultured on *Botrytis cinerea* and isolated by Baermann funnel technique [5]. The harvested PWNs were rinsed 3 times with distilled water and then prepared as an aqueous suspension for further experiments.

### Fungi Culture

*Esteya vermicola* CNU 120806 [3] was a present given by Dr. Chang-Keun Sung and Dr. Chun-yan Wang, Chungnam National University, Korea. The fungus was cultured on potato sucrose agar (PSA: potato exudate 200 g, sucrose 20 g and agar 15 g in 1 liter water) in Petri plates (7 cm diam.) at 26°C for 8 days.

### Chemicals

The chemicals used in GC-MS analyses and silicone tube method (STM) bioassays include α-pinene (98%, J&K Scientific Ltd.), β-pinene (98%, J&K Scientific Ltd.), camphor (95%, J&K Scientific Ltd.), dichloromethane (99%, Aladdin Chemistry Co., Ltd.), and ethanol absolute (99.8%, Aladdin Chemistry Co., Ltd.).

### VOCs Collection, Fractionation, and Analysis

VOCs from *E. vermicola* were collected using the super Q absorbent (80–100 mesh, Alltech Associates Inc., Deerfield, IL, USA), fractionized by GC, and analyzed by GC-MS [6], [7], [8]. The fungus *E. vermicola* was cultured on 200 ml PSA medium in glass tube (30 cm long, 6 cm inner diam.) at 26°C for 10 d. An absorbent glass tube (external and inner diam. were 6 mm and 4 mm respectively) containing 50 mg of Super Q was connected to the outlet of the fungus tube by a Teflon tube. Charcoal-purified air was drawn over the fungus and absorbent tubes at a rate of 300 ml/min by a vacuum pump (Nanjing Rongshide Trade, Nanjing, P. R. China). VOCs were collected for 1 h and then eluted with 150 µl of dichloromethane from Super Q tube into individual 2 ml vial (CNW technology GmbH, Germany) and the resulting extracts were stored at - 20°C until use [6], [8]. VOCs from PSA medium were also collected using the same method as above, but without *E. vermicola*.

Volatile collections from the fungus and PSA medium were repeated three times. Extracts were combined separately, and then concentrated to ∼10 µl with nitrogen stream. One microliter of each extract was analyzed by an Agilent 7890A GC or by an Agilent 7890A connected to an Agilent 5973C mass spectrometer. A 30 m HP-5 ms (0.25 mm internal diam., 0.25 µm film thickness for GC-MS and 0.32 mm internal diam., 0.25 µm film thickness for GC) capillary column (Agilent Technologies Inc., CA, USA) was used with splitless mode. The oven temperature was programmed from an initial temperature of 40°C, and then increased to 280°C at a rate of 5°C/min, held for 10 min.

**Figure 1:**
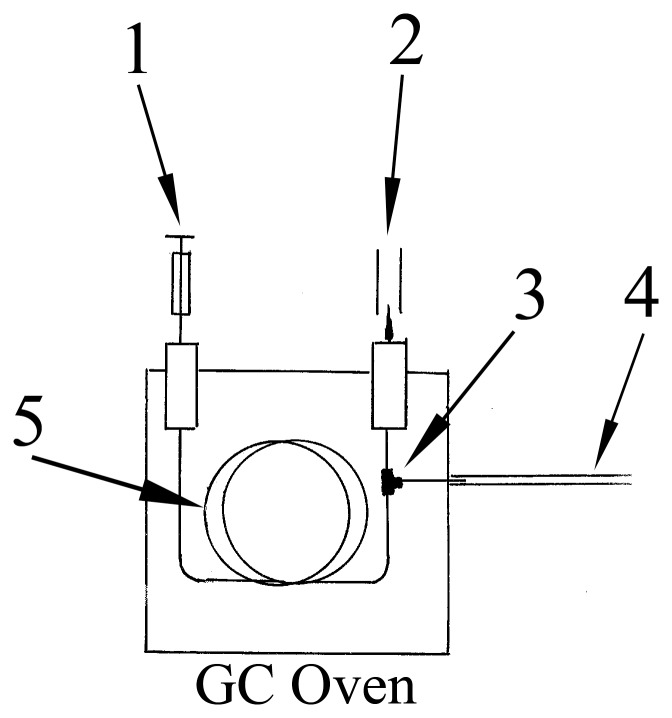
Diagram showing the method to collect VOCs by adding a Y column splitter to the end of GC capillary column: (1) injector; (2) FID; (3) Y column splitter; (4) glass capillary collection tube; and (5) capillary column.

The setup for the GC fractionation is similar to GC-electroantennogram. The end of capillary column was split by a deactivated Y splitter (Agilent Technologies Inc., CA, USA) with its one end to the GC flame ionization detector and the other end as a outlet to the outside using the same type of the column segments (Figure 1). After injecting 6 µl extract from *E. vermicola*, VOCs components were collected during certain retention time periods from GC outlet using glass capillary tubes (20 cm long, 1 mm internal diam.). Each fraction was rinsed with 10 µl of dichloromethane from capillary tube into a small vial and stored at - 20°C until use. Components of interested compounds from extracts were identified by GC-MS based on comparison of mass spectra with the NIST 08 MS library [6], [8] and retention times of synthetic standards. Quantification of α-pinene, β-pinene, and camphor was performed by GC using external standard method.

### Attraction Bioassays

**Figure 2:**
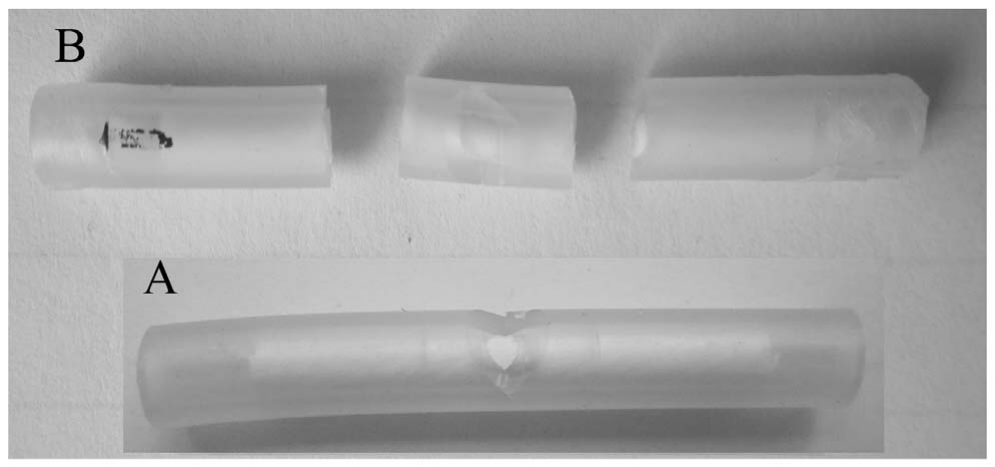
Silicone tube method (STM) for bioassay: (A) the silicon tube with a small hole in the middle and (B) the silicon tube were cut into three segments after the bioassay.

In order to evaluate the activity of VOCs collected from *E. vermicola*, a simple bioassay method that we refer as silicone tube method (STM) was developed (Figure 2). Advantage of STM is that the small internal space in STM greatly increases the concentration of VOCs treatment comparing with Petri dish method that is usually used to study attractive VOCs from microbes to nematodes by most researchers [6], resulting in enhanced sensitivity of bioassay.

The main part of bioassay apparatus was consisted of a 5 cm long transparent silicone tube (6 mm external diam., 3 mm internal diam., TOGOHK International Industrial Co., Ltd). Filter paper of the suitable size was rolled into a 3 cm long rod, gently put into the silicon tube, and kept at the middle of the tube with rooms of 1 cm for samples in the both ends. The paper rod was moistened with 140 µl distilled water. An apical hole (∼0.6 mm diam.) was made in the middle of the silicone tube (Figure 2A), allowing a suspension of mixed aged nematode to be introduced into the filter paper rod inside.

A small filter paper disc (3 mm diam.) containing one microliter of fungal volatile extract, one microliter of volatile extract from PSA medium, one microliter of volatile extract from different fungal GC fractions, or certain amounts of individual synthetic compound or blend (α-pinene, β-pinene, and camphor) was placed into one end of the silicone tube, and the control disc containing dichloromethane solvent was placed into the other end. Two ends of the tube were sealed with parafilm tape (Pechiney Plastic Packaging Company), and the tube was incubated in the dark at 26°C for 0.5 h to establish a concentration gradient of VOCs. After 10 µl of nematode suspension (∼200 mixed aged individuals) was applied to the central opening of the tube then tube was kept horizontally for 10 h in the dark at 26°C. Six replicates were prepared for each treatment. The silicone tube was cut into three segments (left: 2 cm, middle: 1 cm, right: 2 cm, Figure 2B). The nematodes in the two 2 cm segments for the treatment and control were isolated with the Baermann funnel technique [5] respectively and the numbers of the nematode in both of the treatment and control segments were counted and recorded respectively.

### Statistical Analyses

To reduce the deviation of nematodes added in the tubes, the nematode numbers obtained from two segments of the treatment and the control were transferred to percentage to perform the statistical analyses. All of the bioassay data were analyzed by independent samples group *t* test with SPSS 13.0 software.

## Results

### Bioassay of the VOCs from *E. vermicola* Mycelium

The VOCs collected from *E. vermicola* and PSA medium were bioassayed by STM. The result showed that the VOCs collected from *E. vermicola* were significantly attracted more nematodes than the control (p<0.01). However, VOCs from PSA medium did not show attractive activity to attract nematodes (p = 0.126) (Table 1). This indicates that active components to attract PWNs are only associated with the VOCs collected from mycelium of *E. vermicola.*

**Table 1:** Number of PWN attracted by VOCs from *E. vermicola* and PSA medium by STM.

VOCs	Treatment (mean ± S.D.)	Control (mean ± S.D.)	Percentage of the treatment	Percentage of the control	P value
*E. vermicola*	55.67±18.15	15.33±6.51	79.84% ±0.02*	21.16% ±0.02	0.000*
PSA medium	18.67±4.73	21.33±4.16	46.51% ±0.08	53.49% ±0.08	0.126

* Statistically different from control (P<0.01).

### GC Fractionations of the VOCs from *E. vermicola* Mycelium and Activity Bioassay

VOCs collected from *E. vermicola* mycelium were fractionized by GC and four fractions were collected at different retention time periods, which were subsequently bioassayed with the nematodes using STM. Bioassay results showed that the potentially active components appeared in middle fractions with retention time from 4–5.7 min (p<0.01) and 5.7–10 min (p<0.05). Other fractions with retention times from 2.6–4 min (p = 0.102), and 10–18 min (p>0.05) did not show significantly attracting activities comparing with solvent control (Table 2).

**Table 2:** Number of PWN attracted to *E. vermicola* VOCs fractions collected from the GC outlet by STM.

Retention time	Treatment (mean ± S.D.)	Control (mean ± S.D.)	Percentage of the treatment	Percentage of the control	P value
2.6–4 min	8.33±4.04	13.33±4.04	37.87% ±0.14	62.13% ±0.14	0.102
4–5.7 min	32.67±13.32	4.33±2.52	88.8% ±0.02**	11.2% ±0.02	0.000**
5.7–10 min	21.00±18.25	15.67±12.66	56.84% ±0.04*	43.16% ±0.04	0.017*
10–18 min	19.67±13.58	14.67±6.11	54.01% ±0.12	45.99% ±0.12	0.464

* Statistically different from control (P<0.05);

** Statistically different from control (P<0.01).

### GC-MS Analyses of the VOCs from *E. vermicola* and the PSA Medium

**Figure 3:**
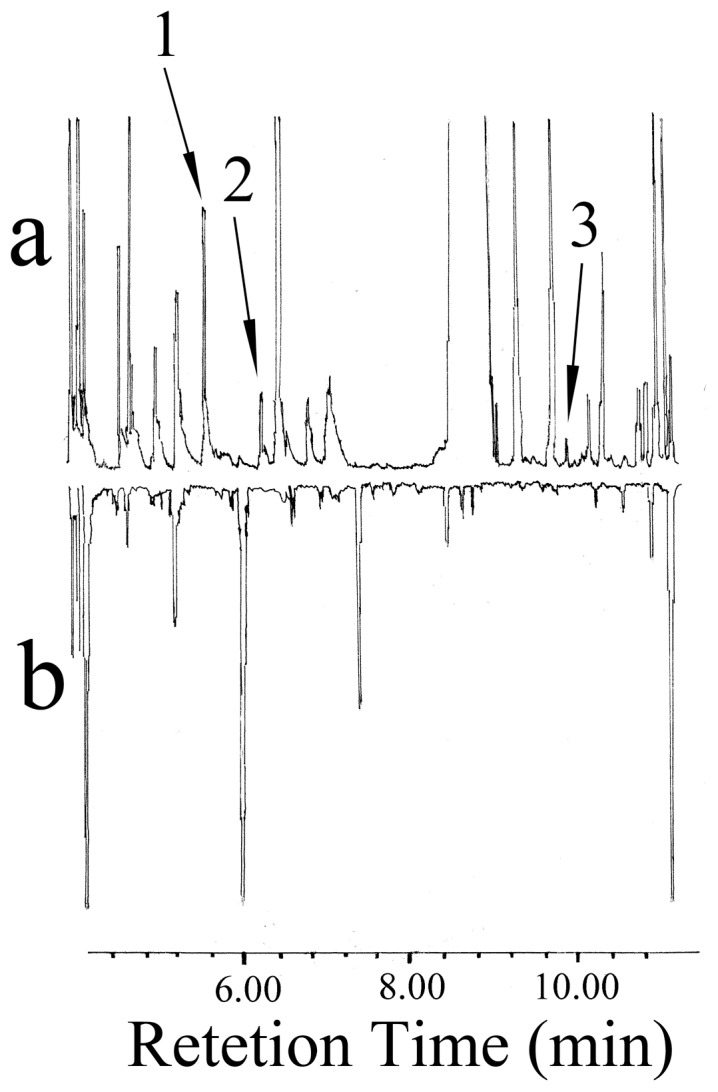
Reconstructed GC-MS total ion chromatograms of the volatile organic compounds (VOCs) from *E. vermicola* and the PSA medium: (a) VOCs collected from *E. vermicola* using the super Q absorbent and (b) VOCs collected from the PSA medium using the super Q absorbent. Identification of peaks: 1, α-pinene; 2, β-pinene; and 3, camphor.

GC-MS analyses of the VOCs from *E. vermicola* and the PSA medium indicated that three compounds were only associated with *E. vermicola* in an approximate ratio of 28: 12: 1 (Figure 3). They were identified as α-pinene, β-pinene, and camphor by comparison of mass spectra with the GC-MS library (Table 3). The identity of these three compounds was also confirmed by GC retention times with synthetic standards.

**Table 3:** Three active components identified by GC-MS from VOCs of *E. vermicola*.

No.	Retention time (min)	Compound	Relative percentage (%)	CAS
1	5.495	α-pinene	0.28%	80-56-8
2	6.164	β-pinene	0.12%	127-91-3
3	9.357	camphor	0.01%	76-22-2

### Bioassays of the Synthetic Compounds

Synthetic α-pinene, β-pinene, and camphor, as well as a blend of the three chemicals were bioassayed with different doses using STM. Our results indicated that three individual compounds and a blend at natural ratio (28: 12: 1) significantly attracted the PWN at appropriate doses (Table 4). The minimum doses to attract PWN for α-pinene, β-pinene, camphor, and blend were 50 ng, 12 ng, 1.6 ng, and 41 ng respectively (p<0.05). No significant synergistic effect was observed when a three component blend was bioassayed at tested concentrations.

**Table 4:** Number of PWN attracted to the individual and the blends of synthetic VOCs by STM.

Compound	Dose in the treatment	Treatment (mean ± S.D.)	Control (mean ± S.D.)	Percentage of the treatment	Percentage of the control	P value
α-pinene	50 ng	16.67±3.06	9.67±2.52	63.57% ±0.03**	36.43% ±0.03	0.001**
α-pinene	28 ng	8.00±3.46	7.00±7.00	53.33% ±0.29	46.67% ±0.29	0.514
β-pinene	12 ng	8.33±3.51	4.33±2.52	65.79% ±0.04**	34.21% ±0.04	0.000**
β-pinene	6 ng	12.33±14.47	11.67±6.51	51.39% ±0.25	48.61% ±0.25	0.560
camphor	1.6 ng	14.67±8.33	9.00±10.86	69.07% ±0.13**	30.93% ±0.13	0.001**
camphor	1 ng	9.00±2.65	11.00±1.00	44.00% ±0.09	55.00% ±0.09	0.232
α-pinene, β-pinene, camphor (28: 12: 1)	41 ng***	7.00±6.38	2.75±3.1	71.56% ±0.21*	28.44% ±0.21	0.026*
α-pinene, β-pinene, camphor (28: 12: 1)	20.5 ng***	6.50±3.21	3.00±12.85	67.06% ±0.40	32.94% ±0.40	0.214

* Statistically different from control (P<0.05);

** Statistically different from control (P<0.01);

*** Total amount of the three chemicals.

## Discussion

Our research results demonstrated that the nematophagous fungus, *E. vermicola*, emitted VOCs to entice PWN (Table 1 and 2), confirming that *E. vermicola* living mycelia produced volatile compounds for PWN attraction [4]. The active compounds from *E. vermicola* VOCs (common pine volatiles: two monoterpenes, α-pinene, β-pinene; and one terpenoid, camphor) were identified (Figure 3, Table 3). The activity of identified compounds has been confirmed by STM bioassay using synthetic compounds (Table 4). To reduce the bioassay time, the middle 1 cm of the tube was cut off and only nematodes in other two segments were counted. That is reason why the numbers of nematodes in the treatment and control were relatively low comparing with total loading. Although it is also possible that the doses of individual synthetic compound and blend used may not be enough to elicit maximal attractive activity and some minor active component may be missing, the synthetic individual and blend tested resulted in up to 71% PWN attraction in our STM bioassay. It supports our hypothesis that attractive volatile compounds from *E. vermicola* should be related to the PWN hosts.

The compounds, α-pinene and β-pinene, have been reported as VOCs components from the larvae and adult of PWN vector beetle *M. alternatus* and its host pine trees, *P. massoniana* and dying *P. thunbergii*, to attract PWN [6], [9], [10], [11]. Yun et al. reported that α-pinene, β-pinene, and camphor were highly attractive to PWN and camphor showed significantly higher attractiveness to the PWNs among the three chemicals in the PWN-infected pine tree log bioassay [12]. More interestingly, our research results demonstrated that *E. vermicola* resorted to host deception, in which this living predaceous fungus enticed PWN by producing VOCs (α-pinene, β-pinene, and camphor) that mimicked the scent of PWN host pine tree for nutrient.

The nematophagous fungi are natural enemies of nematodes because they can trap living nematodes and the attraction intensity increased with increasing dependence of the fungi on nematodes for nutrients [13]. The ability to use nematodes as an additional nutrient source provides nematophagous fungi with a nutritional advantage, therefore, they have been recently used to control the animal-parasitic nematodes in livestock [14], [15], [16], [17], [18] as biological agents. Zhen Wang et al. found that *E*. *vermicola* survived in resin and other chemicals secreted by pine trees, and reproduced with new lunate conidia to parasitize other migratory PWNs in host trees [19]. Our findings are of significances in better understanding parasitic mechanism of the fungus and the co-evolution in the two organisms and will aid management of the pine wilt disease.

To date, the most researchers use Petri dish method to study attractive VOCs to nematodes from microbes [6], [12]. The STM bioassay provides a simple and effective way to assist identification of attractive VOCs to nematodes from microbes. New STM not only enables us to identify attractive compounds to the PWN from VOCs of *E. vermicola*, but also provides a more sensitive and efficient bioassay method in studying the behavior of nematodes. Further work to identify the non-volatile compounds from *E. vermicola* is under way.
